# Discrepancies Between Classic and Digital Epidemiology in Searching for the Mayaro Virus: Preliminary Qualitative and Quantitative Analysis of Google Trends

**DOI:** 10.2196/publichealth.9136

**Published:** 2017-12-01

**Authors:** Mohammad Adawi, Nicola Luigi Bragazzi, Abdulla Watad, Kassem Sharif, Howard Amital, Naim Mahroum

**Affiliations:** ^1^ Padeh and Ziv Hospitals Bar-Ilan Faculty of Medicine Bar-Ilan University Zafat Israel; ^2^ Postgraduate School of Public Health Department of Health Sciences University of Genoa Genoa Italy; ^3^ Edinburgh Medical Missionary Society Nazareth Hospital Nazareth Israel; ^4^ Sackler Faculty of Medicine Tel Aviv University Tel Aviv Israel; ^5^ Department of Medicine ‘B’ Zabludowicz Center for Autoimmune Diseases Sheba Medical Center Tel Hashomer Israel

**Keywords:** digital health, digital epidemiology, emerging viruses, Mayaro virus, arboviruses, epidemiology, epidemiological monitoring

## Abstract

**Background:**

Mayaro virus (MAYV), first discovered in Trinidad in 1954, is spread by the Haemagogus mosquito. Small outbreaks have been described in the past in the Amazon jungles of Brazil and other parts of South America. Recently, a case was reported in rural Haiti.

**Objective:**

Given the emerging importance of MAYV, we aimed to explore the feasibility of exploiting a Web-based tool for monitoring and tracking MAYV cases.

**Methods:**

Google Trends is an online tracking system. A Google-based approach is particularly useful to monitor especially infectious diseases epidemics. We searched Google Trends from its inception (from January 2004 through to May 2017) for MAYV-related Web searches worldwide.

**Results:**

We noted a burst in search volumes in the period from July 2016 (relative search volume [RSV]=13%) to December 2016 (RSV=18%), with a peak in September 2016 (RSV=100%). Before this burst, the average search activity related to MAYV was very low (median 1%). MAYV-related queries were concentrated in the Caribbean. Scientific interest from the research community and media coverage affected digital seeking behavior.

**Conclusions:**

MAYV has always circulated in South America. Its recent appearance in the Caribbean has been a source of concern, which resulted in a burst of Internet queries. While Google Trends cannot be used to perform real-time epidemiological surveillance of MAYV, it can be exploited to capture the public’s reaction to outbreaks. Public health workers should be aware of this, in that information and communication technologies could be used to communicate with users, reassure them about their concerns, and to empower them in making decisions affecting their health.

## Introduction

Mayaro virus (MAYV) was first discovered in Trinidad in 1954 and isolated by Charles Anderson and collaborators from the blood of 5 febrile forest workers [1-3]. MAYV is similar to the chikungunya and Semliki Forest viruses, being a linear, positive-sense, single-stranded, enveloped RNA virus, more specifically an arbovirus of the family Togaviridae and of the genus *Alphavirus*) [4]. MAYV is generally spread by the *Haemagogus* mosquito [5], but it can also be spread by *Aedes aegypti* and *Aedes albopticus* mosquitoes, which appear to be competent vectors as well [6,7]. Rubber workers are particularly at risk of developing MAYV infection.

In the past, small and occasional outbreaks have been described mainly in the Amazon basin of Brazil and a few other parts of South America [8-11]. The first epidemics were reported in 1955 in Brazil and Bolivia [12]. The reemergence of MAYV is cause for great concern for both public health organizations and communities. Anthropogenic changes in ecosystems and environments, due to a variety of phenomena, including urbanization, globalization and migration, agricultural intensification, and deforestation, together with displacement of populations and invasion of wildlife habitats by humans and domestic animals, are playing a major role in MAYV reemergence. Climate changes, economic downturns, and poverty are further drivers of the reemergence of neglected tropical diseases [13]. Therefore, eliminating mosquito breeding sites constitutes an important preventive measure. Efforts to strengthen and improve pathogen surveillance technologies are also fundamental in programs to control disease.

MAYV infection is a nonfatal and generally self-limiting disease causing arthritis in the knee, ankle, and small joints of the extremities, generalized myalgia, frontal headaches and photophobia, vertigo, nausea and epigastric pain, and chills, followed in two-thirds of patients by a fine maculopapular rash affecting the trunk and the extremities. In some cases, MAYV infection can persist up to 2 months.

In May 2007, an outbreak occurred in Chuquisaca Department, Bolivia, and involved 12 persons [14]. In January 2010, a French tourist, after a 15-day trip in the Amazon forest, Brazil, reported MAYV infection [15]. In 2011, MAYV was diagnosed in a 27-year-old male Swiss tourist returning from Peru [16]. MAYV disease was also imported into the United States by 2 infected people who had visited eastern Peru [17] and, more recently, into the Netherlands by a couple infected during their holidays in Suriname [18].

In June 2010, an outbreak occurred in Venezuela, with 69 cases in Ospino, Portuguesa state, and 2 additional cases in San Fernando de Apure, Apure state, on June 7, for a total of 71 confirmed cases as of June 8 (out of the initially reported 77 cumulative cases) [19]. A single case of MAYV infection in an 8-year-old child with fever and abdominal pain was described in rural Haiti (in the Gressier-Léogâne area, 20 miles west of Port-au-Prince) in 2015 [20].

In conclusion, MAYV has been so far isolated in humans, wild animals, and mosquitoes in Bolivia, Brazil [21,22], Colombia, Costa Rica, French Guiana [23], Guatemala, Guyana, Panama, Peru [24], Suriname, Trinidad, and Venezuela [25].

Given the emerging importance of MAYV [26-28], we here aimed to explore the feasibility of exploiting a Web-based tool for monitoring and tracking cases of MAYV infection.

## Methods

Google Trends (Google Inc) is a freely available, online tracking system that, properly using keyword(s), enables a visualization of hit-search volumes in terms of relative search volumes (RSVs). In more detail, for each keyword or string of keywords, searches can be performed using search term or search topic strategies. With the first option, Google Trends tracks and monitors the exact text typed by the user. The second strategy, instead, consists of an exhaustive and systematic collection of all searches semantically related to the given query. Generally, the second search option results in broader findings.

A Google-based approach seems to be particularly useful to monitor infectious diseases epidemics [29]. Pelat and collaborators [30], as well as Valdivia and Monge-Corella [31], documented the usefulness of using Google Trends in capturing influenza and chickenpox outbreaks. The nowcasting or forecasting approach has been used also for other tropical diseases, such as malaria [32], Ebola [33], West Nile virus [34], and dengue [35].

In this study, we systematically searched Google Trends from its inception (January 2004 through to May 2017), using as keywords “virus Mayaro,” “Mayaro virus,” “virus de Mayaro,” and “virus del Mayaro.” We carried out this investigation according to the guidelines and recommendations put forth by Nuti and coworkers [36].

We built an ad hoc database of cases of MAYV by extensively mining Google, Google Scholar, the scholarly literature (PubMed or MEDLINE, Scopus, Scientific Electronic Library Online, and Latin American and Caribbean Health Sciences Literature), epidemiological alerts (from the US Centers for Disease Control and Prevention, European Centre for Disease Prevention and Control, World Health Organization, and Pan American Health Organization), HealthMap, and ProMED-mail reports.

Since Web searches can be prompted by different external or environmental cues (media coverage, education system, etc), we carried out a multivariate regression analysis according to the following predictive model:

RSV(%) = α × scientific interest + β × epidemiology + γ × media impact + ε.

We measured “scientific interest” by counting the number of MAYV-related articles indexed in PubMed or MEDLINE in the study period, using scientific production as a proxy of the interest of the scientific community toward MAYV; “epidemiology” was the number of confirmed MAYV cases; we assessed “media impact” as the number of MAYV-related news items released in the openly available news aggregator Google News as a proxy of the media coverage and influence over public opinion; and ε is the intercept of the model.

This model was theoretically inspired by the extant scholarly literature on infodemiology and infoveillance [37-40] and, in particular, on Google Trends, as well as by a study by Segev and Baram-Tsabari [41], which systematically investigated different search patterns in terms of the roles of the media and the education system.

We chose the best model according to the goodness-of-fit statistical model.

All statistical analyses were performed with IBM SPSS (version 24.0; IBM Corporation). Figures with a *P* value less than .05 were considered statistically significant.

## Results

**Figure 1:**
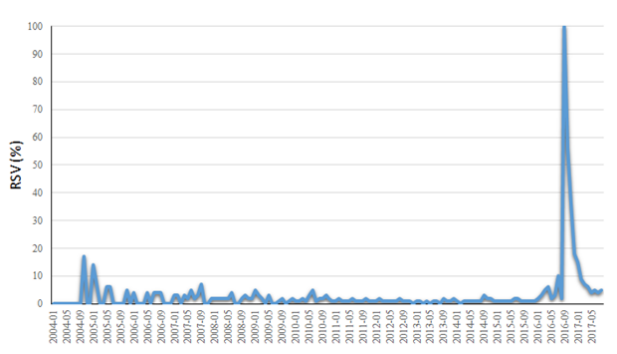
Time trend of Mayaro virus-related Web searches as captured by Google Trends worldwide in the study period (from January 2004 to May 2017). RSV: relative search volume.

Figure 1 shows the MAYV-related RSV trend. A burst in search volumes can be noticed in the period from July 2016 (RSV=13%) to December 2016 (RSV=18%), with a peak in September 2016 (RSV=100%). Before this burst, the average search activity related to MAYV was very low (median 1%).

**Figure 2:**
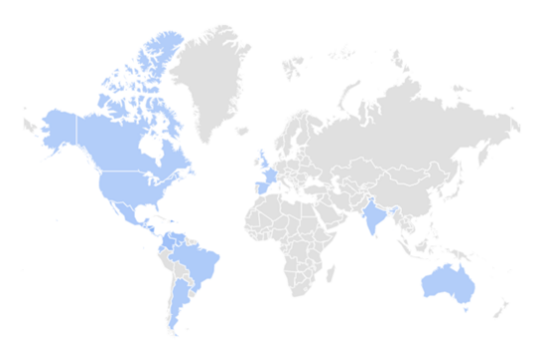
Spatial trend of Mayaro virus-related Web searches as captured by Google Trends worldwide in the study period (from January 2004 to May 2017).

Figure 2 and Table 1 show the countries with major search volumes. MAYV-related queries were concentrated in the Caribbean.

**Figure 3:**
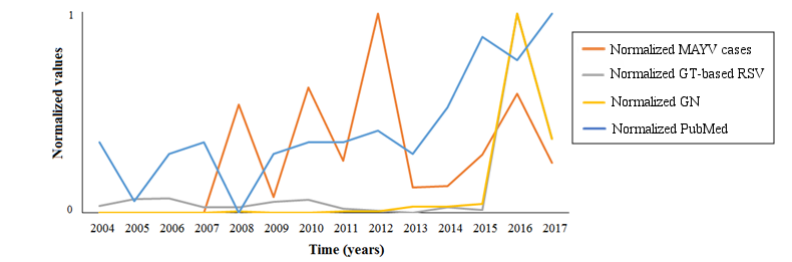
Temporal trends of the different data streams used in the investigation, during the study period (January 2004 to May 2017). GN: Google News; GT: Google Trends; MAYV: Mayaro virus; RSV: relative search volume.

**Figure 4:**
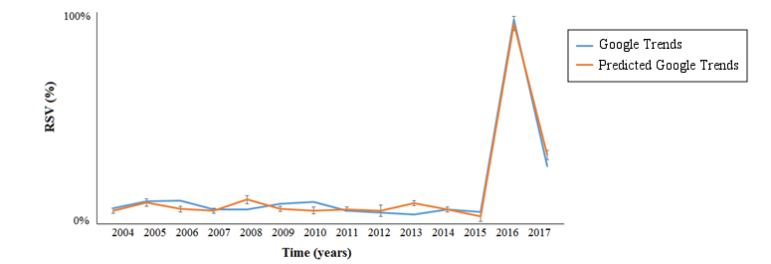
Fit between Google Trends and its prediction according to the best multivariate regression model. RSV: relative search volume.

According to the best multivariate regression model, both scientific interest and media coverage had an impact on seeking behavior, with negative and positive effects, respectively (Table 2,Figure 3,Figure 4).

Textbox 1 lists the top MAYV-related and rising queries. These mainly related to MAYV infection symptoms (eg, fever, influenza-like symptoms), its carriers and vectors of transmission (eg, mosquitoes of the *Aedes* genus), and other similar tropical diseases (eg, yellow fever, malaria, dengue, chikungunya).

Table 3 reports the different models and their goodness-of-fit statistics.

**Table 1:** Countries in which Mayaro virus-related Web queries were concentrated in the study period.

Region	RSV^a^ (%)
Curaçao	100
Dominican Republic	48
Trinidad and Tobago	24
Puerto Rico	22
Nicaragua	20
Honduras	17
El Salvador	15
Martinique	12
Guadalupe	11
Colombia	10
Guatemala	9
Venezuela	8
Jamaica	7
Panama	4
Mexico	3
Ecuador	2
Brazil	2
Costa Rica	2
Argentina	1

^a^RSV: relative search volume.

**Table 2:** Multivariate regression models estimating the impact of different predictors^a^.

Source		Value	SE	T	*P* value	95% CI
**Epidemiology**						
	Intercept	27.313	22.046	1.239	.24	–20.720 to 75.347
	Cases	0.352	0.434	0.811	.43	–0.593 to 1.297
**Epidemiology + media impact**						
	Intercept	14.684	4.313	3.405	.006	5.192 to 24.176
	Cases	0.028	0.086	0.328	.75	–0.160 to 0.217
	Google News	0.718	0.041	17.648	<.001	0.628 to 0.807
**Epidemiology + scientific interest**						
	Intercept	–12.942	31.445	–0.412	.69	–82.152 to 56.267
	PubMed	5.628	3.331	1.689	.12	–1.704 to 12.960
	Cases	0.341	0.404	0.846	.42	–0.547 to 1.230
**Epidemiology + media impact + scientific interest**						
	Intercept	27.358	5.288	5.173	<.001	15.575 to 39.141
	Confirmed cases	0.002	0.065	0.038	.970	–0.143 to 0.148
	Google News	0.783	0.037	20.936	<.001	0.699 to 0.866
	PubMed	–1.931	0.635	–3.043	.01	–3.345 to –0.517

^a^The epidemiological predictor is given by the number of confirmed Mayaro virus cases; the bibliometric predictor is given by the number of articles published in PubMed or MEDLINE; the media predictor is given by the number of news items concerning the Mayaro virus.

**The top Mayaro virus-related and rising queries as captured by Google Trends in the study period.**

Top related queries

- Virus
- Symptoms
- Disorder
- Fever
- Mayaro, Trinidad
- Chikungunya
- Virus Zika
- Dengue
- Aedes
- Mosquito
- Yellow fever mosquito
- Virus Usutu
- Colombia
- Venezuela
- Haiti
- Yellow fever
- World Health Organization
- Midge
- Vaccine
- Alphavirus
- Encephalitis
- Influenza
- Centers for Disease control and prevention
- Malaria

Rising queries

- Symptoms
- Disorder
- Fever
- Mayaro, Trinidad
- Chikungunya
- Virus Zika
- Dengue
- Aedes
- Mosquito
- Yellow fever mosquito
- Virus Usutu
- Colombia
- Venezuela
- Haiti
- Yellow fever
- World Health Organization
- Midge
- Vaccine
- Alphavirus
- Encephalitis
- Influenza
- Centers for Disease control and prevention
- Epidemic outbreak

**Table 3:** Goodness-of-fit statistics for the different multivariate regression models.

Fitting parameter	Epidemiology	Epidemiology + media impact	Epidemiology + scientific interest	Epidemiology + scientific interest + media impact
*R*^2^	.052	.968	.247	.983
Adjusted *R*^2^	–.027	.962	.110	.978
Mean square of errors	3898.689	145.088	3376.945	82.855
Root mean square of errors	62.439	12.045	58.111	9.102
Mean absolute percentage error	119.495	36.418	122.541	29.780
Durbin-Watson statistic	1.615	1.447	2.489	1.796
Mallows C_p_ coefficient	2.000	3.000	3.000	4.000
Akaike information criterion	117.599	72.306	116.370	65.129
Schwarz Bayesian criterion	118.878	74.224	118.287	67.685
Amemiya prediction criterion	1.264	0.050	1.163	0.030

## Discussion

### Principal Findings

The geospatial and temporal epidemiology of MAYV as captured by Google Trends did not reflect the real-world epidemiology of MAYV. For example, Google Trends did not capture several epidemic outbreaks (briefly overviewed in the introduction), including one of the largest MAYV outbreaks, which occurred in northwestern Venezuela in 2010, in a rural village, with 77 cases and 19 individuals confirmed seropositive [19].

Moreover, areas in which MAYV is known to circulate and spread were scarcely represented in terms of search volumes, while areas in which MAYV has been isolated only recently and has never been seen before, such as in the Caribbean, were overrepresented. In the case of MAYV, Google Trends seemed to capture more of the public reaction to MAYV reemergence in terms of worries and concerns, rather than the real-world epidemiological figures. In the last years, there have been concerns about the ability of MAYV to mutate and adapt to new environments, spreading from South America to North America and other countries, and emerging as a “new Ebola,” a “new Zika,” or the “next chikungunya,” thus giving rise to a new public health emergency [26]. The last decades have been characterized by the reemergence of several arboviruses and, above all, by unexpected changes in their clinical history, such as that Zika virus infection can result in neurological disorders and fetal microcephaly [42]. This has led to public concerns and worries, amplified, in their turn, by imbalanced and distorted media coverage. In the case of MAYV, as can be seen using Google News, a freely available aggregator of media news, the report of a single case of an infected child attracted more media attention than all other MAYV cases in South America, as well as receiving more tweets and videos. On the other hand, this single case report suggested that MAYV-related scenarios are changing or could be further changing in the near future, making the possibility of finding MAYV in urban locations carried by anthropophilic insect vectors more concrete [43].

MAYV-related queries concerned above all the symptoms of the infectious disease. No query was related to preventive measures (either environmental or personal hygiene) that could be taken to reduce and mitigate its spreading. This undoubtedly constitutes a major gap in knowledge that public health workers and officials should fill, by providing and disseminating adequate information.

Specifically concerning Google Trends, its validity in complementing classic epidemiological and surveillance techniques and approaches has recently been questioned by some scholars. While there is a relatively huge body of literature reporting the feasibility of exploiting Google Trends in the field of digital epidemiology [44,45], some scholars have criticized Google Trends, showing that it may be inaccurate in some cases, such as that of influenza surveillance [46,47]. Google Flu Trends, based on Google Trends for the epidemiological monitoring of influenza, has been publicly withdrawn, following different criticisms (Google Dengue Trends met a similar fate). On the other hand, Santillana and collaborators [48] demonstrated that some techniques, inspired by data assimilation techniques, supervised machine learning, and artificial intelligence, could be applied to improve the reliability of Google Flu Trends.

Cervellin and colleagues [49], on the contrary, found that Google Trends was a scarcely reliable epidemiological tool in a variety of clinical settings, ranging from renal colic or epistaxis to mushroom poisoning, meningitis, *Legionella pneumophila* pneumonia, and Ebola fever.

Similarly, Tran and coworkers [50], searching Google Trends for “suicide” from 2004 to 2010 in the United States and Switzerland, and from 2004 to 2012 in Germany and Austria, found that Google Trends was not able to forecast national suicide rates.

Our study showed that media coverage resulted in seeking behavior and that this impact can be quantified using a multivariate regression model. This is in line with the findings of Segev and Baram-Tsabari [41], who found that ad hoc events or current concerns correlated better with media coverage than did general or well-established scientific terms. Indeed, MAYV, being an emerging virus, represents a relatively overlooked research field and only recently has caused severe public health problems and concerns, which have resulted both in increased media attention and coverage (as shown by Google News) and increased interest from the scientific community (as shown by the bibliometric data), as well as in higher search volumes from the public (as shown by Google Trends).

### Strengths and Limitations

This study has some strengths, including the systematic search of MAYV-related queries and the novelty of the investigation, being the first, to the best of our knowledge, to address the topic of the relationship between MAYV and information and communication technologies (ICTs).

However, our investigation is not without limitations. The major drawback is that Google Trends returns relative, normalized values instead of providing scholars with absolute, raw figures that can be further handled, refined, and statistically processed. A second shortcoming is the digital divide, in that Google Trends captures only that segment of the population that actively uses the new ICTs and devices. However, this segment is constantly growing and increasing. A third drawback is that, when using Google Trends, only Google-based searches and queries can be tracked and monitored. On the other hand, Google is the most commonly used search engine worldwide.

### Conclusions

MAYV, an arthropod-borne virus, has always circulated in South America. Its recent appearance in the Caribbean has been a source of concern, which has resulted in a burst of Internet queries. While Google Trends cannot be used to perform real-time epidemiological surveillance of MAYV, it can be exploited to capture the public reaction to outbreaks, in terms of worries, and knowledge needs and gaps [51]. Public health workers and officials should be aware that they can use Google Trends to easily track and monitor public reaction and popular perceptions, and use ICTs to communicate with users, reassure them about their concerns, and empower them in making decisions affecting their health [51-54].

Further studies in the field are needed, especially using other ICTs and social media or networks, such as Twitter, Facebook, or Instagram, as well as carrying out a content analysis of MAYV-related digital material. Moreover, techniques for correcting and revising Google Trends should be systematically explored, for example, correlating Web searches with environmental parameters (such as rainfall, temperature, or weather), which are well known to have an impact on the epidemiology of neglected tropical infectious diseases.

## Abbreviations

ICTs: information and communication technologies
MAYV: Mayaro virus
RSV: relative search volume
